# Antibacterial Activity of 1-[(2,4-Dichlorophenethyl)amino]-3-Phenoxypropan-2-ol against Antibiotic-Resistant Strains of Diverse Bacterial Pathogens, Biofilms and in Pre-clinical Infection Models

**DOI:** 10.3389/fmicb.2017.02585

**Published:** 2017-12-22

**Authors:** Valerie Defraine, Laure Verstraete, Françoise Van Bambeke, Ahalieyah Anantharajah, Eleanor M. Townsend, Gordon Ramage, Romu Corbau, Arnaud Marchand, Patrick Chaltin, Maarten Fauvart, Jan Michiels

**Affiliations:** ^1^Centre of Microbial and Plant Genetics, University of Leuven, Leuven, Belgium; ^2^Center for Microbiology, Vlaams Instituut voor Biotechnologie, Leuven, Belgium; ^3^Pharmacologie Cellulaire et Moléculaire, Louvain Drug Research Institute, Université catholique de Louvain, Brussels, Belgium; ^4^Oral Science Research Group, Glasgow Dental School, University of Glasgow, Glasgow, United Kingdom; ^5^Institute of Healthcare Policy and Practice, University of West of Scotland, Paisley, United Kingdom; ^6^CISTIM Leuven vzw, Leuven, Belgium; ^7^Centre for Drug Design and Discovery, Leuven, Belgium; ^8^Department of Life Sciences and Imaging, Smart Electronics Unit, imec, Leuven, Belgium

**Keywords:** antibacterials, *P. aeruginosa*, ESKAPE pathogens, anti-persister therapies, antibiotic resistance

## Abstract

We recently described the novel anti-persister compound 1-[(2,4-dichlorophenethyl)amino]-3-phenoxypropan-2-ol (SPI009), capable of directly killing persister cells of the Gram-negative pathogen *Pseudomonas aeruginosa*. This compound also shows antibacterial effects against non-persister cells, suggesting that SPI009 could be used as an adjuvant for antibacterial combination therapy. Here, we demonstrate the broad-spectrum activity of SPI009, combined with different classes of antibiotics, against the clinically relevant ESKAPE pathogens *Enterobacter aerogenes, Staphylococcus aureus, Klebsiella pneumoniae, Acinetobacter baumannii, P. aeruginosa, Enterococcus faecium* and *Burkholderia cenocepacia* and *Escherichia coli*. Importantly, SPI009 re-enabled killing of antibiotic-resistant strains and effectively lowered the required antibiotic concentrations. The clinical potential was further confirmed in biofilm models of *P. aeruginosa* and *S. aureus* where SPI009 exhibited effective biofilm inhibition and eradication. *Caenorhabditis elegans* infected with *P. aeruginosa* also showed a significant improvement in survival when SPI009 was added to conventional antibiotic treatment. Overall, we demonstrate that SPI009, initially discovered as an anti-persister molecule in *P. aeruginosa*, possesses broad-spectrum activity and is highly suitable for the development of antibacterial combination therapies in the fight against chronic infections.

## Introduction

Antibiotic resistance is rapidly increasing in the majority of nosocomial pathogens, complicating the effective treatment of bacterial infections and transforming once easily cured diseases into serious human health threats (European Centre for Disease Prevention and Control, 2013; O’Neill, 2016). Although selection for resistance in microorganisms is inevitable, the widespread and excessive use of antibiotics allowed pathogens to efficiently adapt to these stressful conditions, resulting in the occurrence of extensively drug-resistant and pan-drug resistant strains (Livermore, 2004; Fischbach and Walsh, 2009). In an attempt to guide research and development toward the most critical pathogens, the World Health Organization (WHO) recently published their ‘global priority list,’ containing 12 bacterial pathogens that raise particular concern (WHO, 2017). Among these are the so-called ESKAPE pathogens, *Enterococcus faecium, Staphylococcus aureus, Klebsiella pneumoniae, Acinetobacter baumannii, P. aeruginosa*, and *Enterobacter* spp., which efficiently evade antibiotic treatment and represent new paradigms in pathogenesis, transmission, and resistance (Rice, 2008). Together, this select group of bacteria is responsible for most of the hospital-acquired infections and, despite increasing research efforts, therapeutic options remain scarce (Bassetti et al., 2013; Pendleton et al., 2013). Greatly contributing to the difficult treatment of these bacterial infections is the presence of non-growing persister cells. These phenotypic variants show a reduced metabolic activity, are able to withstand intensive antibiotic treatment, and when antibiotic pressure drops, are capable of restoring the bacterial population, causing recurrence of infection (Fauvart et al., 2011; Van den Bergh et al., 2017). Persistence is widely acknowledged as a major culprit of treatment failure in chronic and biofilm infections and recent research has identified the persister fraction as a possible reservoir for the development of resistance (Lewis, 2007; Cohen et al., 2013). Effective elimination of persister cells could significantly improve patient outcomes, but their small numbers and the apparent redundancy in persister mechanisms greatly hampers the development of targeted anti-persister therapies.

We recently reported the identification of a novel anti-persister molecule capable of directly killing persister cells of *P. aeruginosa* (Liebens et al., 2017). SPI009 was identified in a screening of 23,909 small molecules for compounds that decrease the persister fraction of *P. aeruginosa* in combination with the conventional antibiotic ofloxacin. In the present study, we explore the activity of SPI009 in several additional pathogens and demonstrate broad spectrum activity and the ability to sensitize resistant strains. Furthermore, SPI009 was shown to retain activity in different biofilm models and is capable of significantly improving antibiotic efficacy both in *in vitro* and *in vivo* infection models. Overall, these results further increase the clinical potential of SPI009 and offer compelling perspectives for the use of SPI009 as an adjuvant in effective antimicrobial therapies.

## Materials and Methods

### Bacterial Strains, Human Cell Lines, *C. elegans*, and Culture Conditions

Bacterial strains used in this study are listed in **Table 1**. All strains were cultured in 1:20 diluted Trypticase Soy Broth (1/20 TSB) at 37°C shaking at 200 rpm. For solid medium, TSB was supplemented with 1.5% agar. Human THP-1 cell lines were cultivated in RPMI-1640 medium containing 10% fetal calf serum at 37°C with 5% CO_2_. The *C. elegans* AU37 strain [g*lp-4(bn2); sek-1(km4)*] was obtained from the Caenorhabditis Genetics Center (CGC) and maintained according to standards (Stiernagle, 2006). The following antibacterials were used: ofloxacin, ciprofloxacin, rifampicin, polymyxin B, vancomycin (Sigma–Aldrich), and 1-[(2,4-dichlorophenethyl)amino]-3-phenoxypropan-2-ol (SPI009; CD3) with concentrations indicated throughout the text.

**Table 1:** Strains used in this study.

Strain	Description	Source or reference
*P. aeruginosa* PA14	Wild type; UBCPP-PA14	Pierre Cornelis; Lee et al., 2006
*P. aeruginosa* PAO1	Wild type	Dieter Haas (ETH)
*P. aeruginosa* PA62	Broncho-pulmonary clinical isolate OFX^R^, CIP^R^, GEN^R^, AMK^R^, ATM^R^, TIC^R^, PIP^R^, TZP^R^, CAZ^R^, FEP^R^	Françoise van Bambeke (UCL)
*P. aeruginosa* 9BR	Clinical isolate, PBM^R^, MEM^R^, CIP^R^, and FEP^R^, CAZ^R^, or TZP^R^	Bob Hancock; Boyle et al., 2012
*E. aerogenes*	ATCC 13048 (KCTC 2190)	Shin et al., 2012
*S. aureus* Rosenbach 1844	Wild type, methicillin resistant, ATCC 33591	BCCM/LGM bacterial collection; Conlon et al., 2013
*K. pneumoniae*	ATCC 13883	Arivett et al., 2015
*A. baumannii*	RUH134	Jean-Paul Pirnay; Merabishvili et al., 2014
*E. faecium*	LMG 8148	Descheemaeker et al., 1997
*B. cenocepacia* K56-2	LMG 18863	Van Acker et al., 2013
*E. coli* BW25113	F^-^, Δ(araD-araB)567, ΔlacZ4787(::rrnB-3), ^-^, rph-1, Δ(rhaD-rhaB)568, *hsd*R514	Baba et al., 2006

*Resistance profiles determined according to EUCAST MIC breakpoints (European Committee on Antimicrobial Susceptibility Testing, 2017). OFX, ofloxacin; CIP, ciprofloxacin; GEN, gentamicin; AMK, amikacin; ATM, aztreonam; TIC, ticarcillin; PIP, piperacillin; TZP, piperacillin-tazobactam; CAZ, ceftazidime; FEP, cefepime; PBM, polymyxin B; MEM, meropenem*.

### Antibacterial Assays

Antibacterial assays were performed on different clinically relevant pathogens as previously described (Liebens et al., 2017). Briefly, stationary phase cultures were treated for 5 h with 17 or 34 μg/mL of SPI009 alone or in combination with an appropriate antibiotic to assess anti-bacterial and anti-persister effects, respectively. To evaluate activity against resistant strains, stationary phase cultures were treated for 5 h with 1x, 4x, and 8x MIC concentrations of the respective antibiotic; 17 or 34 μg/mL SPI009 or the combination of both. After treatment, cells were washed and viability was assessed via plating.

### Quantification of Biofilm Formation and Eradication after Treatment with SPI009

Overnight cultures of *P. aeruginosa* PA14 WT or *S. aureus* ATCC 33591 were diluted 1:100 in 1/20 TSB medium supplemented with 2% DMSO (carrier control) or increasing concentrations of SPI009 (4.25–68 μg/mL). Biofilms were grown for 24 h at 37°C on the bottom of a polystyrene 96-well plate, non-shaking. Medium and free-living cells were removed and the biofilms were washed, scraped off and passed five times through a syringe (0.5 mm × 1.6 mm) to disrupt any cell clumps and obtain single cells (Hermans et al., 2011). Appropriate dilutions made in 1x PBS were plated on solid TSB agar plates to assess biofilm growth under different conditions.

To explore the biofilm eradicating effects of SPI009, overnight cultures of *P. aeruginosa* PA14 WT or *S. aureus* ATCC 33591 were diluted 1:100 in 1/20 TSB medium and incubated for 24 h at 37°C (non-shaking). Mature biofilms were treated for 5 h with 2% DMSO and increasing concentrations of SPI009 (8.5–136 μg/mL) at 37°C, non-shaking, after which the remaining biofilms were processed as described above.

### Chronic Wound Model

A three-dimensional wound biofilm model was used, as previously described (Townsend et al., 2016). *P. aeruginosa* coated cellulose matrices, obtained after 2 h of adhesion (1 × 10^6^ cells/mL), were placed onto the hydrogels after which 3D biofilm development was allowed for 24 h at 37°C. Mature biofilms were treated for 24 h with DMSO (1%), 10 μg/mL ofloxacin, 34 and 69 μg/mL of SPI009 or the combination of ofloxacin and SPI009. Any non-adherent cells were removed by rinsing after which biomass was removed by sonication at 35 kHz for 10 min and DNA was extracted. Samples were prepared as previously described and viability-based qPCR using *P. aeruginosa* specific primers F- GGGCGAAGAAGGAAATGGTC and R- CAGGTGGCGTAGGTGGAGAA was used to determine live and total fractions of biofilm cells under different treatment conditions (Smith et al., 2016). Standard curves were used to convert the obtained qPCR values to colony forming estimates (CFEs), after which log_10_-transformed values were used for statistical analysis, as described below. All experiments were carried out in triplicate, each containing three technical repeats.

### Intracellular Infection Model

Infection of human THP-1 cells was performed as described previously, with minor modifications (Buyck et al., 2013). Since a newly synthesized batch of SPI009 was used for this experiment, cytotoxicity assessment via an LDH enzyme assay was repeated for the THP-1 cell line, as previously described (Liebens et al., 2017). After THP-1 infection with *P. aeruginosa* PAO1 and subsequent removal of any non-phagocytozed or adherent bacteria, ciprofloxacin and SPI009 were added in final concentrations of, respectively, 0–20 μg/mL and 6.8 or 10.2 μg/mL. After 5 h of treatment, eukaryotic cells were collected in three consecutive centrifugation steps and complete cell lysis was obtained by sonication (10 s). Lysates were used for bacterial CFU counting and determination of protein content by Lowry’s assay (Bio-Rad DC protein assay kit; Bio-Rad laboratories, Hercules, CA, United States). For analysis of surviving bacterial cells, CFU data were divided by corresponding protein content for normalization.

### *C. elegans* Toxicity Testing and Survival Assay

AU37 nematodes were synchronized as previously described (Porta-de-la-Riva et al., 2012) to obtain L4 worms suitable for toxicity and infection assays (Briers et al., 2014). Larvae obtained after bleaching were plated onto solid NGM-OP50 agar plates and incubated at 25°C during 2 days to allow development of the worms to the L4 stage. Worms were transferred to fresh NGM agar plates containing OP50 (toxicity testing and uninfected control) or PA14 (infection) for an additional 24 h at 25°C.

To evaluate toxicity of SPI009 L4 nematodes grown on OP50 were transferred to 12-well plates (20–30 worms/well) containing different concentrations of SPI009 (8.5–136 μg/mL) in 1.5 mL NGM:M9 (1:4). Controls consisted of untreated worms and DMSO (2% and 20%). For the infection assay, adult worms were allowed to feed on NGM-PA14 plates for 24 h, after which residual bacteria were removed and nematodes were divided over a 12-well plate (20–30 worms/well). Different treatments were prepared in 1.5 mL NGM:M9 (1:4) and consisted of an untreated control, 1.56 μg/mL ciprofloxacin (5x MIC), 8.5 μg/mL of SPI009 and the combination of ciprofloxacin and SPI009. As an additional control, uninfected worms were included. For both assays, worms were incubated at 25°C and survival was scored visually for 6 days.

### Statistical Analysis

Unless mentioned otherwise, all statistical analyses were performed on log_10_-transformed data using GraphPad Prism software (version 6.01). Bacterial survival after different treatments was compared to the untreated or antibiotic control using a one-way ANOVA (α = 0.05), with Dunnett’s correction for multiple comparisons. Statistical comparison of mono- and combination treatment in resistant strains was done using a two-way ANOVA (α = 0.05) with Tukey correction for multiple comparisons. Statistical analysis of the *in vivo C. elegans* data was done by means of a log-rank test using GraphPad Prism.

## Results

### SPI009 Shows Broad-Spectrum Activity against Different Clinically Relevant Bacterial Species

**FIGURE 1:**
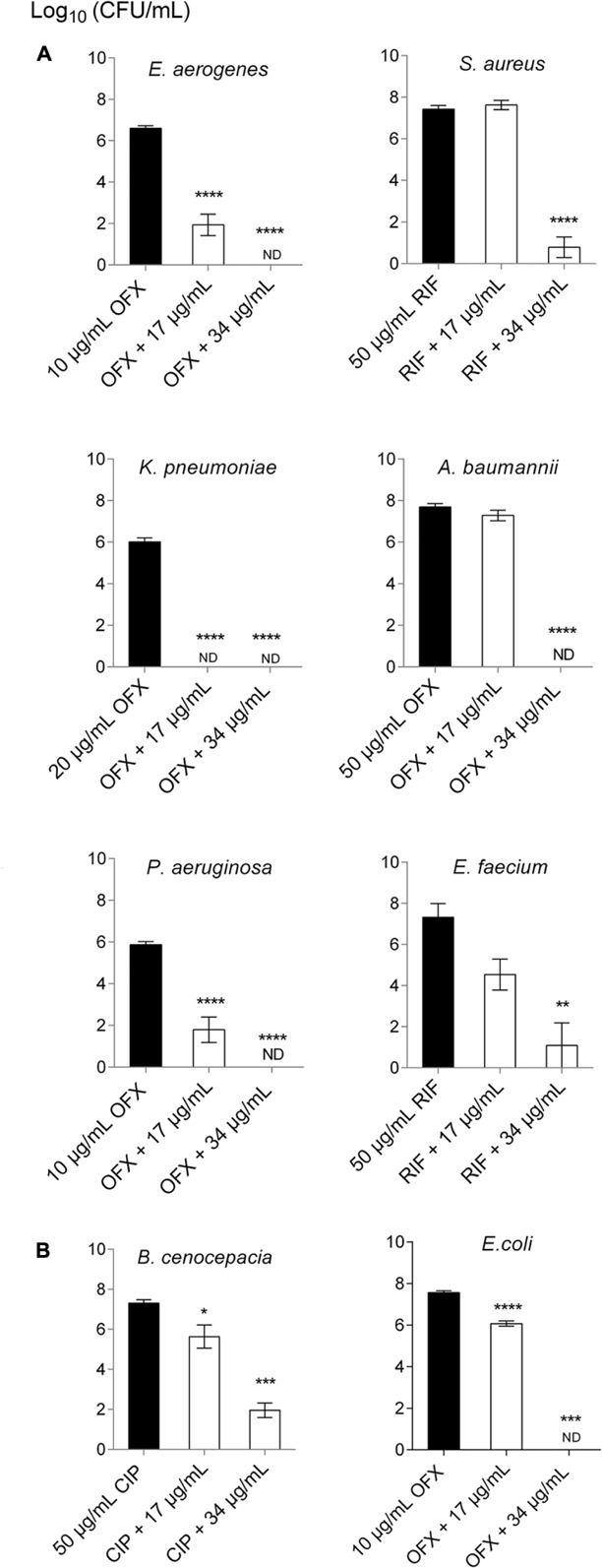
SPI009 possesses broad-spectrum activity against different clinically important pathogens. 200 μL volumes of stationary phase cultures of **(A)** ESKAPE pathogens *E. aerogenes, S. aureus, K. pneumoniae, A. baumannii, P. aeruginosa*, and *E. faecium* and **(B)** *B. cenocepacia* and *E. coli* were treated for 5 h with the combination of a conventional antibiotic; ofloxacin (OFX), ciprofloxacin (CIP), or rifampicin (RIF) and 17 or 34 μg/mL SPI009. Black bars represent the antibiotic and white bars the combination of antibiotic with SPI009. Results are the mean of at least three independent experiments with error bars depicting SEM values. One-way ANOVA with Dunnett’s correction for multiple comparisons was used to detect significant differences to the antibiotic control with ^∗^*P* ≤ 0.05, ^∗∗^*P* ≤ 0.01, ^∗∗∗^*P* ≤ 0.001, ^∗∗∗∗^*P* ≤ 0.0001. ND, not detected.

The activity of SPI009 was previously assessed in *P. aeruginosa* PA14 and several clinical isolates where combination with ofloxacin significantly decreased the persister fraction in all strains tested (Liebens et al., 2017). In the present study, we challenged a panel of clinically relevant species, including the ESKAPE pathogens (**Figure 1A**), *B. cenocepacia* and *E. coli* (**Figure 1B**). For each species appropriate concentrations of a conventional antibiotic used in the clinic were selected to allow only persister cells to survive (Supplementary Figure S1). Combination of the antibiotic with 17 μg/mL SPI009 significantly decreased the number of surviving bacteria for five of the eight species with reductions in CFU ranging between 1.5 ± 0.1 and 6.0 ± 0.2 log units and complete eradication of *K. pneumoniae*. Addition of 34 μg/mL completely eradicated the bacterial cultures of five of the eight species tested and resulted in significant 6.6 ± 0.5 log, 6.2 ± 1.3 log, and 5.4 ± 0.5 log reductions in bacterial survival for *S. aureus*, *E. faecium*, and *B. cenocepacia*, respectively. No reduction in survival is observed after treatment with 17 μg/mL for either of the Gram-positive species, *E. faecium* and *S. aureus*. These results suggest that the latter two species, and the Gram-negative *B. cenocepacia*, are slightly less sensitive toward the combination therapy. *K. pneumoniae* proved the most susceptible species toward SPI009. Overall, the obtained results further support the antibacterial effect of SPI009 and reveal a broad-spectrum activity.

### SPI009 Sensitizes Antibiotic-Resistant Strains

**FIGURE 2:**
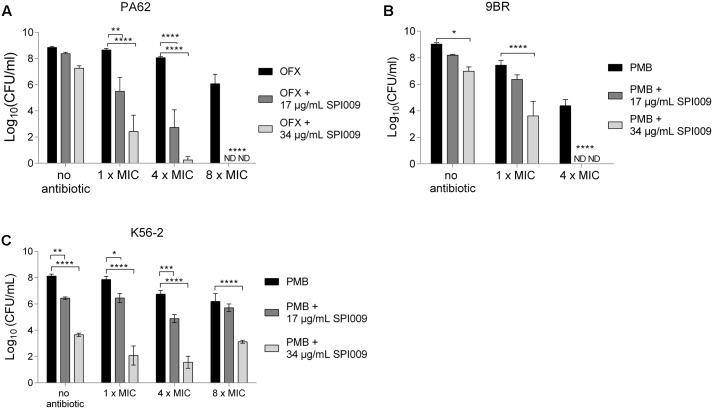
SPI009 re-enables the treatment of (multi)drug-resistant strains. Stationary phase cultures of **(A)** *P. aeruginosa* PA62 (OFX^R^), **(B)** *P. aeruginosa* 9BR (PMB^R^) and **(C)** *B. cenocepacia* K56-2 (PMB^R^) were treated for 5 h with 1x MIC, 4x MIC, and 8x MIC concentrations of the respective antibiotic alone and in combination with 17 or 34 μg/mL of SPI009. Data points represent the average of at least three biological repeats. SEM values are shown as error bars. Statistical analysis was done by means of two-way ANOVA (α = 0.05) with Tukey correction for multiple comparisons and ^∗^*P* ≤ 0.05, ^∗∗^*P* ≤ 0.01, ^∗∗∗^*P* ≤ 0.001, ^∗∗∗∗^*P* ≤ 0.0001; ND, not detected.

To investigate the possible use of SPI009 as an adjuvant in antibacterial combination therapies, several (multi)drug-resistant strains were treated with 1x, 4x, and 8x MIC concentrations of the antibiotic, alone and in combination with SPI009. While SPI009 alone did not cause a significant decrease in survival of the ofloxacin resistant *P. aeruginosa* PA62, addition of 17 or 34 μg/mL of SPI009 significantly reduced the number of surviving cells by 5.3 ± 0.9 and 7.8 ± 0.9 log units at 4x MIC of ofloxacin while combination with 8x MIC completely eradicated the bacterial culture (**Figure 2A**). In comparison, treatment with ofloxacin alone caused 0.8 ± 0.9 log and 2.8 ± 0.9 log decreases in surviving cells at concentrations of 4x MIC and 8x MIC, respectively.

A similar trend was observed in the polymyxin B resistant *P. aeruginosa* 9BR (**Figure 2B**). Here, addition of the antibiotic alone had a slightly greater effect but combination with SPI009 still significantly improved the treatment and 17 μg/mL of SPI009 successfully eradicated the entire bacterial culture in combination with 4x MIC of polymyxin B. A somewhat smaller effect was observed in the polymyxin B resistant *B. cenocepacia* strain K56-2, for which addition of 17 μg/mL and 34 μg/mL SPI009 to 4x MIC polymyxin B resulted in significant 4.9 ± 0.5 and 5.2 ± 0.5 log decreases in survival. Combinations with higher concentrations of polymyxin B (8x MIC) did not further decrease the number of surviving cells (**Figure 2C**). The obtained results clearly demonstrate the effective use of SPI009 as an adjuvant for antibacterial therapy thereby facilitating the treatment of different antibiotic-resistant strains. Furthermore, SPI009 retains activity in multidrug-resistant strains, revealing the lack of cross-resistance. Importantly, resensitization of resistant strains could restore the effectiveness of established antibiotics.

### Biofilm Inhibition and Eradication Effects of SPI009

**FIGURE 3:**
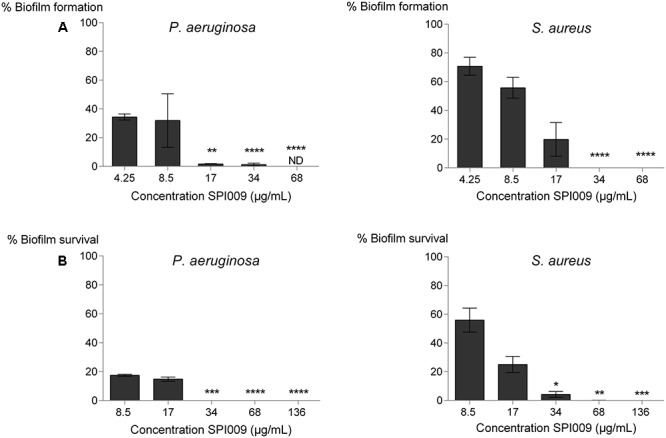
Anti-biofilm effects of SPI009 in *P. aeruginosa* and *S. aureus.* Increasing concentrations of SPI009 (4.25–136 μg/mL) and a DMSO control were added for **(A)** 24 h to 1:100 diluted cultures or **(B)** 5 h to 24-h-old biofilms in 96-well microtiter plates to assess biofilm inhibition and eradication, respectively. After treatment, biofilms were washed, disturbed and plated out to calculate the number of surviving cells. Data points represent the percentage of surviving cells relative to the untreated control as an average of at least three biological repeats, each containing three technical repeats. Error bars depict SEM values. Statistical significance was calculated on log_10_ transformed CFU counts using a one-way ANOVA with Dunnett’s correction for multiple comparisons. ^∗^*P* ≤ 0.05, ^∗∗^*P* ≤ 0.01, ^∗∗∗^*P* ≤ 0.001, ^∗∗∗∗^*P* ≤ 0.0001. ND, not detectable.

To assess biofilm inhibiting properties of SPI009 in *P. aeruginosa* and *S. aureus*, biofilm growth was allowed in the presence of increasing concentrations of SPI009 (**Figure 3A**). Analysis of the obtained results clearly show an effective inhibition of biofilm growth in both *P. aeruginosa* and *S. aureus.* For *P. aeruginosa*, a steep increase in inhibitory activity was observed at concentrations above 8.5 μg/mL, resulting in 1.8 ± 0.5 log and 2.4 ± 0.4 log decreases at 17 or 34 μg/mL SPI009, respectively, and complete inhibition of biofilm growth at 68 μg/mL. *S. aureus* showed a more gradual decrease in biofilm formation with 34 μg/mL and 68 μg/mL resulting in significant 6.2 ± 0.6 log and 6.4 ± 0.6 log decreases in biofilm formation, respectively. These results clearly demonstrate the potent biofilm inhibiting activity of SPI009 for both Gram-negative and Gram-positive model pathogens.

To explore biofilm eradication, SPI009 was added to mature biofilms and survival was assessed after 5 h of treatment. For *P. aeruginosa* the lower concentrations (8.5 and 17 μg/mL) caused a decrease in biofilm survival of about 0.8 log units (**Figure 3B**). Doses of 34 μg/mL or higher significantly decreased the number of surviving biofilms cells, resulting in 4.2 ± 0.6; 6.2 ± 0.6; and 6.6 ± 0.6 log reductions. In comparison, 10 μg/mL of the conventional antibiotic ofloxacin caused a significant 4.5 ± 1 log decrease in the number of surviving biofilm cells (Supplementary Figure S2A). For *S. aureus*, the treatment of mature biofilms with lower concentrations of SPI009 proved slightly less effective than for *P. aeruginosa*. Treatment with higher concentrations did cause extensive damage, resulting in significant decreases in biofilm survival ranging between 2.5 ± 0.7 and 5.4 ± 0.6 log. For the 96-well biofilm models used in this study, the combination of SPI009 with a conventional antibiotic did not further decrease the number of surviving cells as compared to mono-treatment with SPI009 (Supplementary Figure S2). Overall, SPI009 shows potent activity in biofilms of both Gram-negative and Gram-positive species and is capable of significantly inhibiting biofilm formation and decreasing survival of mature biofilms.

### SPI009 Reduces Bacterial Load in a Chronic Wound Model

**FIGURE 4:**
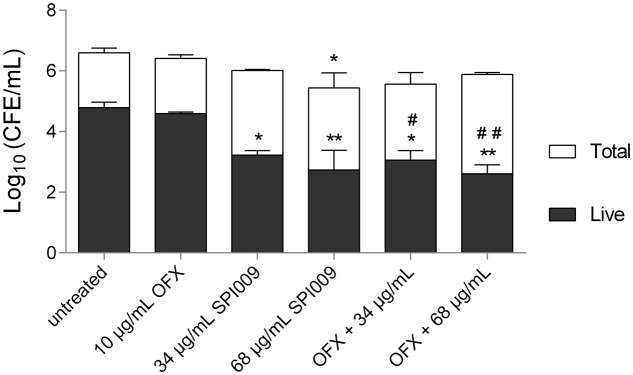
Effect of SPI009 in a chronic wound model. Mature biofilms, grown on hydrogels supporting a cellulose matrix, were treated for 24 h with 10 μg/mL ofloxacin, 34 or 68 μg/mL of SPI009 alone or in combination with ofloxacin. Washed biofilms were removed by sonication and viability was assessed by means of Live/Dead PCR. Values are shown as the log_10_ transformation of colony forming estimates (CFEs), as determined for three biological repeats each containing three technical repeats. Error bars represent SEM values. Significant differences relative to untreated control are depicted by ^∗^, ^#^ represent significant differences relative to ofloxacin treatment with ^∗^/^#^ *P* ≤ 0.05, ^∗∗^/^##^ *P* ≤ 0.01, as determined by means of one-way ANOVA with Dunnett’s correction.

After confirming the biofilm eradication capacity of SPI009 in a standard biofilm set-up, a more clinically relevant model was used to assess the clinical potential of SPI009 as a topical antibacterial treatment. Using a porous cellulose matrix placed upon a moist hydrogel allowed the growth of a complex, three-dimensional hydrated structure, effectively mimicking biofilms in a chronic wound environment (Townsend et al., 2016; Kean et al., 2017). Assessment of viability was performed by means of live/dead quantitative PCR (**Figure 4**). For the viable cells, treatment with increasing concentrations of SPI009 alone resulted in significant 1.6 ± 0.5 log (34 μg/mL) and 2.0 ± 0.5 log (68 μg/mL) decreases in the number of surviving cells. The obtained results confirm the biofilm eradication capacity of SPI009, both as an antimicrobial and as part of a combination therapy, and this in a more complex, realistic biofilm environment.

### SPI009 Potentiates Antibiotic Activity in an Intracellular Infection Model

**FIGURE 5:**
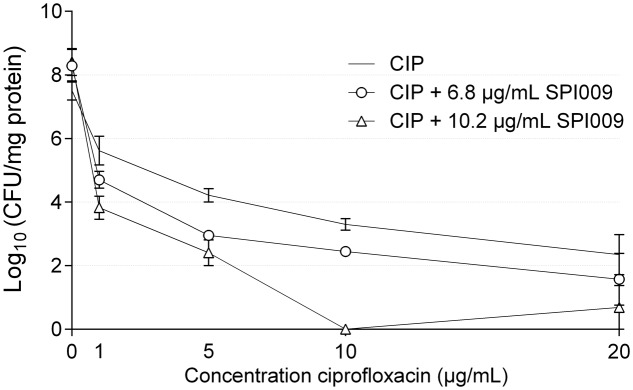
Addition of SPI009 enhances antibiotic treatment of intracellular infections. Human THP-1 cells were infected for 5 h with *P. aeruginosa* PAO1 (MOI 1:10) after which extracellular bacteria were removed and cells were treated with increasing concentrations of ciprofloxacin, alone or in combination with 6.8 μg/mL or 10 μg/mL of SPI009. Data points represent the average of at least two biological repeats, each containing three technical repeats. Error bars depict SEM values. Statistical comparison between ciprofloxacin treatment and the different combination treatments was done by means of one-way ANOVA testing with correction for multiple comparisons (Dunnett) (α = 0.05).

Next, the anti-persister and antibacterial activities of SPI009 were verified in a recently developed *P. aeruginosa* intracellular infection model (Buyck et al., 2013). Human THP-1 cells were infected with PAO1 cells (MOI 10) and treated for 5 h with different concentrations of ciprofloxacin, alone or in combination with 6.8 or 10.2 μg/mL of SPI009. Concentrations of SPI009 were chosen to be well below the determined IC_50_ value of 24.5 ± 1.36 μg/mL. After treatment, both the number of surviving PAO1 cells and the amount of eukaryotic proteins present was assessed, as this can provide information about the possible toxic effect of the different treatments and the infecting bacteria. While treatment with SPI009 alone caused non-significant decreases of 0.78 ± 0.7 and 0.89 ± 0.7 log units in surviving bacteria, addition of SPI009 to ciprofloxacin greatly improved the antibacterial effect for all concentrations tested and this in a dose-dependent manner (**Figure 5**). Maximal antibacterial activity for the combination therapy with 10.2 μg/mL of SPI009 occurs at ciprofloxacin concentrations of 10 μg/mL, resulting in complete eradication of the bacterial culture. Moreover, all combinations tested significantly reduced the bacterial load as compared to ciprofloxacin alone. Combination treatment with 6.8 μg/mL SPI009 showed a maximal 0.78 ± 0.6 log decrease as compared to antibiotic alone at a ciprofloxacin concentration of 20 μg/mL. These results clearly show that SPI009 can effectively penetrate the eukaryotic cell membrane, without causing extensive damage, to eradicate the intracellular *P. aeruginosa* infection.

### SPI009 Combination Therapy Significantly Improves *in Vivo* Survival

Since the antibacterial effect of SPI009 was demonstrated extensively *in vitro*, a next step was to assess the effect of this new compound in an *in vivo C. elegans* gut infection model. Toxicity testing of SPI009 in *C. elegans* revealed minor levels of toxicity at 68 μg/mL and >80% killing at 136 μg/mL (Supplementary Figure S3), excluding these concentrations from further experiments. Analysis of the different DMSO concentrations suggests that the observed toxicity is mainly caused by increasing concentrations of the solvent.

**FIGURE 6:**
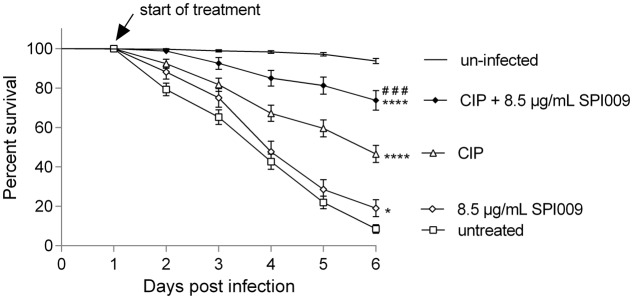
SPI009 combination therapy enhances *C. elegans* survival in a PA14 WT infection assay. Δ*glp-4(bn2)/*Δ*sek-1(km4) C. elegans* worms were infected with *P. aeruginosa* by feeding them on NGM-PA14 WT plates for 24 h. Worms were treated with 8.5 μg/mL SPI009 (open diamonds), 1.56 μg/mL ciprofloxacin (5x MIC; open triangles) or the combination of SPI009 with ciprofloxacin (filled circles). Untreated worms (open squares) and uninfected worms (solid line) served as controls. Worms were counted daily for 6 days with nematode survival expressed as a percentage relative to the viability on day 1. Data points represent the mean of at least three independent repeats ± SEM. Statistical analysis was performed on Kaplan–Meier plots by means of the log-rank test (α = 0.05). Significant differences to the untreated control are represented by ^∗^, ^#^ represent significant differences to the ciprfloxacin control. ^∗^*P* ≤ 0.05, ^###^*P* ≤ 0.001 and ^∗∗∗∗^ *P* ≤ 0.0001.

Infection of nematodes with PA14 resulted in 91.5% killing within 6 days after the start of infection, confirming the highly virulent nature of the PA14 strain in this model (**Figure 6**). Addition of 8.5 μg/mL of SPI009 alone slightly improved survival but not as good as 5x MIC of ciprofloxacin, resulting in survival rates of 19.0% (*P* = 0.045) and 46.6% (*P* < 0.0001), respectively. However, addition of 8.5 μg/mL of SPI009 to ciprofloxacin greatly increased survival, resulting in 73.8% nematode survival after 6 days. These results show a significant improvement in antibacterial effect of the combination therapy compared to the untreated (*P* < 0.0001) and ciprofloxacin-treated (*P* = 0.0001) controls (Supplementary Table S1). Since low doses of SPI009 can greatly enhance the effect of conventional antibiotic treatment, resulting in more than 73% survival, these results indicate the highly efficient antibacterial and potentiating effect of SPI009 as part of a combination therapy.

## Discussion

Decades of excessive drug prescription, misuse of antimicrobials and extensive agricultural applications have caused a massive increase in drug resistance. Conventional antibiotic therapies are losing the battle against emerging extensively drug-resistant strains, resulting in 25,000 annual deaths in the European Union (European Centre for Disease Prevention and Control, 2009). A group of pathogens raising particular concern are the so-called ESKAPE pathogens, *E. faecium, S. aureus, K. pneumoniae*, *A. baumannii*, *P. aeruginosa*, and *Enterobacter* spp. Responsible for the majority of nosocomial infections, these pathogens show significant rises in resistance rates and are becoming increasingly difficult to treat with currently available antibiotics (Boucher et al., 2009; Pendleton et al., 2013). Since it is becoming alarmingly difficult to identify novel antibiotic targets, combination therapies could provide an alternative strategy for the effective treatment of bacterial infections. When different mode of actions are combined, they can lower the risk of resistance development and extend the life span of currently available antibiotics (Tamma et al., 2012; Gill et al., 2015). However, additional research is needed to assess possible negative effects associated with combination therapies and to determine an optimal combination *in vivo* (Tamma et al., 2012; Pena-Miller et al., 2013). An additional advantage of combination therapies is their potential use in the treatment of persister cells (Cui et al., 2016; Feng et al., 2016; Yang et al., 2016; Gallo et al., 2017; Koeva et al., 2017), a small reservoir of phenotypical variants that tolerate antibiotic treatment and reinitiate bacterial infection when the antibiotic pressure drops. The antibiotic-tolerant phenotype of persister cells contributes to the recalcitrant nature of chronic infections, greatly complicates treatment and increases the chances of resistance development (Lewis, 2007; Fauvart et al., 2011; Michiels et al., 2016).

We recently described the discovery of the propanol-amine derivative SPI009, a novel anti-persister molecule capable of directly killing persister cells of *P. aeruginosa* (Liebens et al., 2017). Most anti-persister molecules described in literature are only active against one or a very limited number of bacterial species, which can be explained by a very specific mode of action or the sensitizing of persister cells to a specific class of antibiotics (Wood, 2015; Van den Bergh et al., 2017). Other examples of small organic compounds capable of directly killing persister cells include the recently described α-bromocinnamaldehyde (Shen et al., 2017), 5-iodoindole (Lee et al., 2016), halogenated phenazines (Garrison et al., 2015) and the nitroimidazole prodrug PA-284 (Singh et al., 2008). In this study, we showed that SPI009 possesses broad-spectrum activity and is capable of significantly decreasing or even eradicating the bacterial culture for all pathogens tested, including the notorious ESKAPE pathogens. In addition, combination therapy of conventional antibiotics with SPI009 allowed the efficient treatment of polymyxin B and ofloxacin resistant strains and could lower the required concentration of antibiotics, thereby enabling their use in resistant strains.

The close relationship between persisters and chronic infections (LaFleur et al., 2006; Mulcahy et al., 2010) is partly caused by their presence in biofilms. The presence of the biofilm matrix is capable of physically protecting the persister cells against the human immune system, thereby enabling the persister cells to resume growth when antibiotic pressure drops and cause recurrence of infection. When compared to other anti-biofilm compounds or conventional antibiotics, SPI009 monotherapy shows a promising anti-biofilm effect, both decreasing biofilm formation and causing a strong reduction in the number of surviving biofilm cells, for both Gram-negative and Gram-positive species. A more clinically relevant biofilm model was obtained by *P. aeruginosa* growth on cellulose matrices and hydrogels, providing a three-dimensional structure and moist environment closely mimicking the environment of a chronically infected wound. In this 3D model, clinical treatments have been shown to have less impact on the viability of biofilms in comparison to traditional 2D models, which are more susceptible to eradication (Townsend et al., 2016; Kean et al., 2017). Therefore this further supports the ability of SPI009 mono-treatment to eradicate cells in a more complex biofilm model and suggests the possible use of SPI009 in the topical treatment of chronically infected wounds. For all biofilm experiments executed, the addition of SPI009 to a conventional antibiotic did not further decrease the biofilm population as compared to SPI009 alone. In comparison to planktonic cultures, where combination therapy with antibiotics strongly enhances the antibacterial effect, the specific lay-out and environment of the bacterial biofilm, including a possibly reduced penetration of antibacterials, could impair the cooperation between both antibacterials.

Besides the biofilm matrix, persister cells have also been shown to use eukaryotic cells to shield themselves from the human immune system. The presence of intracellular persister reservoirs has been confirmed *in vivo* and can be associated with the chronic nature of infections (Buyck et al., 2013; Helaine et al., 2014). The ability of SPI009 to effectively reduce the intracellular bacteria further confirms the potential of SPI009 as an adjuvant in combination therapies. Capable of increasing nematode survival to more than 70% when combined with ciprofloxacin, the *in vivo C. elegans* model further contributes to the clinical potential of SPI009. The *C. elegans* model has been extensively used in the identification and clinical assessment of novel antibacterials and antifungals with ample studies confirming the consistent correlation between toxic effects in *C. elegans* and mammalian models (Hunt, 2017).

## Conclusion

We demonstrated that the anti-persister molecule SPI009 possesses a broad-spectrum antibacterial activity and, taken into account that it can be combined with different classes of antibiotics, shows great potential for the development of case-specific antibacterial combination therapies. The clinical potential of SPI009 was further confirmed by the observation of an excellent anti-biofilm activity, successful eradication of an intracellular infection in human eukaryotes and the significant increase in *C. elegans* survival after treatment with the combination of SPI009 and ciprofloxacin. Additional *in vivo* experiments will be required to assess the future applicability of SPI009 but its excellent activity in antibacterial combination therapies holds great promise.

## Author Contributions

Conceptualization, VD, RC, AM, PC, MF, and JM. Methodology, VD, FVB, GR, MF, and JM. Formal analysis, VD. Investigation, VD, LV, AA, and EMT. Wrote the original draft, VD. Contributed in writing review and editing, VD, FVB, GR, MF, and JM. Visualization, VD. Supervision, MF and JM.

## Conflict of Interest Statement

The authors declare that the research was conducted in the absence of any commercial or financial relationships that could be construed as a potential conflict of interest.

## References

[B1] Arivett B. A. Ream D. C. Fiester S. E. Mende K. Murray C. K. Thompson M. G. (2015). Draft genome sequences of *Klebsiella pneumoniae* clinical type strain ATCC 13883 and three multidrug-resistant clinical isolates. *Genome Announc.* 3:e01385-14. 10.1128/genomeA.01385-14 25593250 PMC4299892

[B2] Baba T. Ara T. Hasegawa M. Takai Y. Okumura Y. Baba M. (2006). Construction of *Escherichia coli* K-12 in-frame, single-gene knockout mutants: the Keio collection. *Mol. Syst. Biol.* 2:2006.0008. 10.1038/msb4100050 16738554 PMC1681482

[B3] Bassetti M. Merelli M. Temperoni C. Astilean A. (2013). New antibiotics for bad bugs: where are we? *Ann. Clin. Microbiol. Antimicrob.* 12:22. 10.1186/1476-0711-12-22 23984642 PMC3846448

[B4] Boucher H. W. Talbot G. H. Bradley J. S. Edwards J. E. Gilbert D. Rice L. B. (2009). Bad bugs, no drugs: no ESKAPE! An update from the Infectious Diseases Society of America. *Clin. Infect. Dis.* 48 1–12. 10.1086/595011 19035777

[B5] Boyle B. Fernandez L. Laroche J. Kukavica-Ibrulj I. Mendes C. M. F. Hancock R. E. W. (2012). Complete genome sequences of three *Pseudomonas aeruginosa* isolates with phenotypes of polymyxin B adaptation and inducible resistance. *J. Bacteriol.* 194 529–530. 10.1128/JB.06246-11 22207740 PMC3256643

[B6] Briers Y. Walmagh M. Van Puyenbroeck V. Cornelissen A. Cenens W. Aertsen A. (2014). Engineered endolysin-based “Artilysins” to combat multidrug-resistant Gram-negative pathogens. *mBio* 5:e1379-14. 10.1128/mBio.01379-14 24987094 PMC4161244

[B7] Buyck J. M. Tulkens P. M. Van Bambeke F. (2013). Pharmacodynamic evaluation of the intracellular activity of antibiotics towards *Pseudomonas aeruginosa* PAO1 in a model of THP-1 human monocytes. *Antimicrob. Agents Chemother.* 57 2310–2318. 10.1128/AAC.02609-12 23478951 PMC3632903

[B8] Cohen N. R. Lobritz M. A. Collins J. J. (2013). Microbial persistence and the road to drug resistance. *Cell Host Microbe* 13 632–642. 10.1016/j.chom.2013.05.009 23768488 PMC3695397

[B9] Conlon B. P. Nakayasu E. S. Fleck L. E. LaFleur M. D. Isabella V. M. Coleman K. (2013). Activated ClpP kills persisters and eradicates a chronic biofilm infection. *Nature* 503 365–370. 10.1038/nature12790 24226776 PMC4031760

[B10] Cui P. Niu H. Shi W. Zhang S. Zhang H. Margolick J. (2016). Disruption of membrane by colistin kills uropathogenic *Escherichia coli* persisters and enhances killing of other antibiotics. *Antimicrob. Agents Chemother.* 60 6867–6871. 10.1128/AAC.01481-16 27600051 PMC5075097

[B11] Descheemaeker P. Lammens C. Pot B. Vandamme P. Goossens H. (1997). Evaluation of arbitrarily primed PCR analysis and pulsed-field gel electrophoresis of large genomic DNA fragments for identification of enterococci important in human medicine. *Int. J. Syst. Bacteriol.* 47 555–561. 10.1099/00207713-47-2-555 9103648

[B12] European Centre for Disease Prevention and Control (2009). *The Bacterial Challenge: Time to React.* Stockholm: ECDC 10.2900/2518

[B13] European Centre for Disease Prevention and Control (2013). *Point Prevalence Survey of Healthcare-associated Infections and Antimicrobial Use in European Acute Care Hospitals 2011–2012.* Stockholm: ECDC.

[B14] European Committee on Antimicrobial Susceptibility Testing (2017). *Breakpoint Tables for Interpretation of MICs and Zone Diameters. Version 7.1 2017.* Available at: http://www.eucast.org

[B15] Fauvart M. De Groote V. N. Michiels J. (2011). Role of persister cells in chronic infections: clinical relevance and perspectives on anti-persister therapies. *J. Med. Microbiol.* 60 699–709. 10.1099/jmm.0.030932-0 21459912

[B16] Feng J. Shi W. Zhang S. Sullivan D. Auwaerter P. G. Zhang Y. (2016). A drug combination screen identifies drugs active against amoxicillin-induced round bodies of *in vitro Borrelia burgdorferi* persisters from an FDA drug library. *Front. Microbiol.* 7:743. 10.3389/fmicb.2016.00743 27242757 PMC4876775

[B17] Fischbach M. A. Walsh C. T. (2009). Antibiotics for emerging pathogens. *Science* 325 1089–1093. 10.1126/science.1176667 19713519 PMC2802854

[B18] Gallo S. W. Ferreira C. A. S. de Oliveira S. D. (2017). Combination of polymyxin B and meropenem eradicates persister cells from *Acinetobacter baumannii* strains in exponential growth. *J. Med. Microbiol.* 66 57–60. 10.1099/jmm.0.000542 28721845

[B19] Garrison A. T. Abouelhassan Y. Kallifidas D. Bai F. Ukhanova M. Mai V. (2015). Halogenated phenazines that potently eradicate biofilms, MRSA persister cells in non-biofilm cultures, and *Mycobacterium tuberculosis*. *Angew. Chem. Int. Ed.* 54 14819–14823. 10.1002/anie.201508155 26480852

[B20] Gill E. E. Franco O. L. Hancock R. E. W. (2015). Antibiotic adjuvants: diverse strategies for controlling drug-resistant pathogens. *Chem. Biol. Drug Des.* 85 56–78. 10.1111/cbdd.12478 25393203 PMC4279029

[B21] Helaine S. Cheverton A. M. Watson K. G. Faure L. M. Matthews S. Holden D. W. (2014). Internalization of *Salmonella* by macrophages induces formation of nonreplicating persisters. *Science* 343 204–208. 10.1126/science.1244705 24408438 PMC6485627

[B22] Hermans K. Nguyen T. L. A. Roberfroid S. Schoofs G. Verhoeven T. De Coster D. (2011). Gene expression analysis of monospecies *Salmonella* Typhimurium biofilms using differential fluorescence induction. *J. Microbiol. Methods* 84 467–478. 10.1016/j.mimet.2011.01.012 21256891

[B23] Hunt P. R. (2017). The *C. elegans* model in toxicity testing. *J. Appl. Toxicol.* 37 50–59. 10.1002/jat.3357 27443595 PMC5132335

[B24] Kean R. Rajendran R. Haggarty J. Townsend E. M. Short B. Burgess K. E. (2017). *Candida albicans* mycofilms support *Staphylococcus aureus* colonization and enhances miconazole resistance in dual-species interactions. *Front. Microbiol.* 8:258 10.3389/fmicb.2017.00258 PMC5322193 28280487

[B25] Koeva M. Gutu A. D. Hebert W. Wager J. D. Yonker L. M. O’Toole G. A. (2017). An anti-persister strategy for the treatment of chronic *Pseudomonas aeruginosa* infections. *Antimicrob. Agents Chemother.* 16:S12. 10.1128/AAC.00987-17 28923873 PMC5700368

[B26] LaFleur M. D. Kumamoto C. A. Lewis K. (2006). *Candida albicans* biofilms produce antifungal-tolerant persister cells. *Antimicrob. Agents Chemother.* 50 3839–3846. 10.1128/AAC.00684-06 16923951 PMC1635216

[B27] Lee D. G. Urbach J. M. Wu G. Liberati N. T. Feinbaum R. L. Miyata S. (2006). Genomic analysis reveals that *Pseudomonas aeruginosa* virulence is combinatorial. *Genome Biol.* 7:R90. 10.1186/gb-2006-7-10-r90 17038190 PMC1794565

[B28] Lee J.-H. Kim Y.-G. Gwon G. Wood T. K. Lee J. (2016). Halogenated indoles eradicate bacterial persister cells and biofilms. *AMB Express* 6:123. 10.1186/s13568-016-0297-6 27921270 PMC5138170

[B29] Lewis K. (2007). Persister cells, dormancy and infectious disease. *Nat. Rev. Microbiol.* 5 48–56. 10.1038/nrmicro1557 17143318

[B30] Liebens V. Defraine V. Knapen W. Swings T. Beullens S. Corbau R. (2017). Identification of 1-((2,4-Dichlorophenethyl)Amino)-3-Phenoxypropan-2-ol, a novel antibacterial compound active against persisters of *Pseudomonas aeruginosa*. *Antimicrob. Agents Chemother.* 61:e00836-17. 10.1128/AAC.00836-17 28630188 PMC5571286

[B31] Livermore D. M. (2004). The need for new antibiotics. *Clin. Microbiol. Infect.* 10 1–9. 10.1111/j.1465-0691.2004.1004.x 15522034

[B32] Merabishvili M. Vandenheuvel D. Kropinski A. M. Mast J. De Vos D. Verbeken G. (2014). Characterization of newly isolated lytic bacteriophages active against *Acinetobacter baumannii*. *PLOS ONE* 9:e104853. 10.1371/journal.pone.0104853 25111143 PMC4128745

[B33] Michiels J. E. Van den Bergh B. Verstraeten N. Michiels J. (2016). Molecular mechanisms and clinical implications of bacterial persistence. *Drug Resist. Updat.* 29 76–89. 10.1016/j.drup.2016.10.002 27912845

[B34] Mulcahy L. R. Burns J. L. Lory S. Lewis K. (2010). Emergence of *Pseudomonas aeruginosa* strains producing high levels of persister cells in patients with cystic fibrosis. *J. Bacteriol.* 192 6191–6199. 10.1128/JB.01651-09 20935098 PMC2981199

[B35] O’Neill J. (2016). *Tackling Drug-resistant Infections Globally: Final Report and Recommendations. The Review on Antimicrobial Resistance.* London: HM Government.

[B36] Pena-Miller R. Laehnemann D. Jansen G. Fuentes-Hernandez A. Rosenstiel P. Schulenburg H. (2013). When the most potent combination of antibiotics selects for the greatest bacterial load: the smile-frown transition. *PLOS Biol.* 11:e1001540. 10.1371/journal.pbio.1001540 23630452 PMC3635860

[B37] Pendleton J. N. Gorman S. P. Gilmore B. F. (2013). Clinical relevance of the ESKAPE pathogens. *Expert Rev. Anti Infect. Ther.* 11 297–308. 10.1586/eri.13.12 23458769

[B38] Porta-de-la-Riva M. Fontrodona L. Villanueva A. Cerón J. (2012). Basic *Caenorhabditis elegans* methods: synchronization and observation. *J. Vis. Exp.* 64:e4019. 10.3791/4019 22710399 PMC3607348

[B39] Rice L. B. (2008). Federal funding for the study of antimicrobial resistance in nosocomial pathogens: no ESKAPE. *J. Infect. Dis.* 197 1079–1081. 10.1086/533452 18419525

[B40] Shen Q. Zhou W. Hu L. Qi Y. Ning H. Chen J. (2017). Bactericidal activity of alpha-bromocinnamaldehyde against persisters in *Escherichia coli*. *PLOS ONE* 12:e0182122. 10.1371/journal.pone.0182122 28750057 PMC5531548

[B41] Shin S. H. Kim S. Kim J. Y. Lee S. Um Y. Oh M.-K. (2012). Complete genome sequence of *Enterobacter aerogenes* KCTC 2190. *J. Bacteriol.* 194 2373–2374. 10.1128/JB.00028-12 22493190 PMC3347075

[B42] Singh R. Manjunatha U. Boshoff H. I. M. Ha Y. H. Niyomrattanakit P. Ledwidge R. (2008). PA-824 kills nonreplicating *Mycobacterium tuberculosis* by intracellular NO release. *Science* 322 1392–1395. 10.1126/science.1164571 19039139 PMC2723733

[B43] Smith K. Collier A. Townsend E. M. O’Donnell L. E. Bal A. M. Butcher J. (2016). One step closer to understanding the role of bacteria in diabetic foot ulcers: characterising the microbiome of ulcers. *BMC Microbiol.* 16:54. 10.1186/s12866-016-0665-z 27005417 PMC4804642

[B44] Stiernagle T. (2006). *Maintenance of C. elegans.* Available at: http://www.wormbook.org 10.1895/wormbook.1.101.1 PMC4781397 18050451

[B45] Tamma P. D. Cosgrove S. E. Maragakis L. L. (2012). Combination therapy for treatment of infections with Gram-negative bacteria. *Clin. Microbiol. Rev.* 25 450–470. 10.1128/CMR.05041-11 22763634 PMC3416487

[B46] Townsend E. M. Sherry L. Rajendran R. Hansom D. Butcher J. Mackay W. G. (2016). Development and characterisation of a novel three-dimensional inter-kingdom wound biofilm model. *Biofouling* 32 1259–1270. 10.1080/08927014.2016.1252337 27841027

[B47] Van Acker H. Sass A. Bazzini S. De Roy K. Udine C. Messiaen T. (2013). Biofilm-grown *Burkholderia cepacia* complex cells survive antibiotic treatment by avoiding production of reactive oxygen species. *PLOS ONE* 8:e58943. 10.1371/journal.pone.0058943 23516582 PMC3596321

[B48] Van den Bergh B. Fauvart M. Michiels J. (2017). Formation, physiology, ecology, evolution and clinical importance of bacterial persisters. *FEMS Microbiol. Rev.* 41 219–251. 10.1093/femsre/fux001 28333307

[B49] WHO (2017). *Global Priority List of Antibiotic-resistant Bacteria to Guide Research, Discovery, and Development of New Antibiotics.* Geneva: WHO.

[B50] Wood T. K. (2015). Combatting bacterial persister cells. *Biotechnol. Bioeng.* 113 476–483. 10.1002/bit.25721 26264116

[B51] Yang S. Hay I. D. Cameron D. R. Speir M. Cui B. Su F. (2016). Antibiotic regimen based on population analysis of residing persister cells eradicates *Staphylococcus epidermidis* biofilms. *Sci. Rep.* 5:18578. 10.1038/srep18578 26687035 PMC4685274

