# Supplementary material for: Antibacterial Activity of 1-[(2,4-Dichlorophenethyl)amino]-3-Phenoxypropan-2-ol against Antibiotic-Resistant Strains of Diverse Bacterial Pathogens, Biofilms and in Pre-clinical Infection Models
Source: Front Microbiol. 2017 Dec 22;8:2585. doi: 10.3389/fmicb.2017.02585 (PMC5744096; doi:10.3389/fmicb.2017.02585)
Supplement: Supplementary file 1 [file Data_Sheet_1.DOCX]

Supplementary Material

Antibacterial activity of 1-((2,4-dichlorophenethyl)amino)-3-phenoxypropan-2-ol against antibiotic-resistant strains of diverse bacterial pathogens, biofilms and in pre-clinical infection models

**Valerie Defraine, Laure Verstraete, Françoise Van Bambeke, Ahalieyah Anantharajah, Eleanor M. Townsend, Gordon Ramage, Romu Corbau, Arnaud Marchand, Patrick Chaltin, Maarten Fauvart, Jan Michiels**

*** Correspondence:** corresponding author: jan.michiels@kuleuven.vib.be

# Supplementary Data

## Supplementary Figures


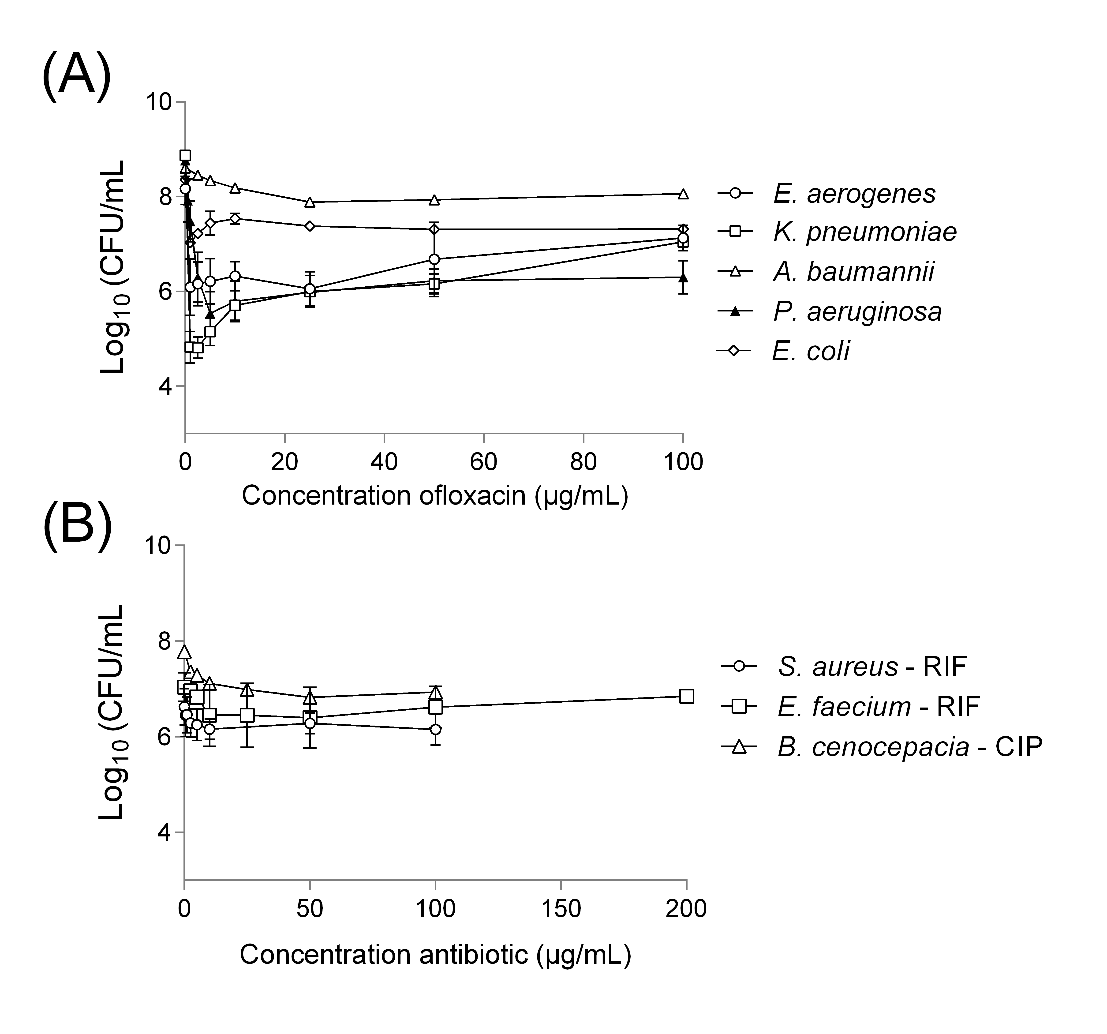


**Figure S1: Determination of the persister plateau.**  Stationary phase cultures of (A) *E. aerogenes* (open circle), *K. pneumoniae* (open square), *A. baumannii* (open triangle), *P. aeruginosa* (filled triangle), *E. coli* (open diamond) and (B) *S. aureus* (open circle), *E. faecium* (open square) and *B. cenocepacia* (open triangle) were treated for 5 hours with increasing concentrations of (A) ofloxacin or (B) rifampicin (RIF) or ciprofloxacin (CIP). Data points represent the means of three independent experiments, with error bars depicting the SEM value.


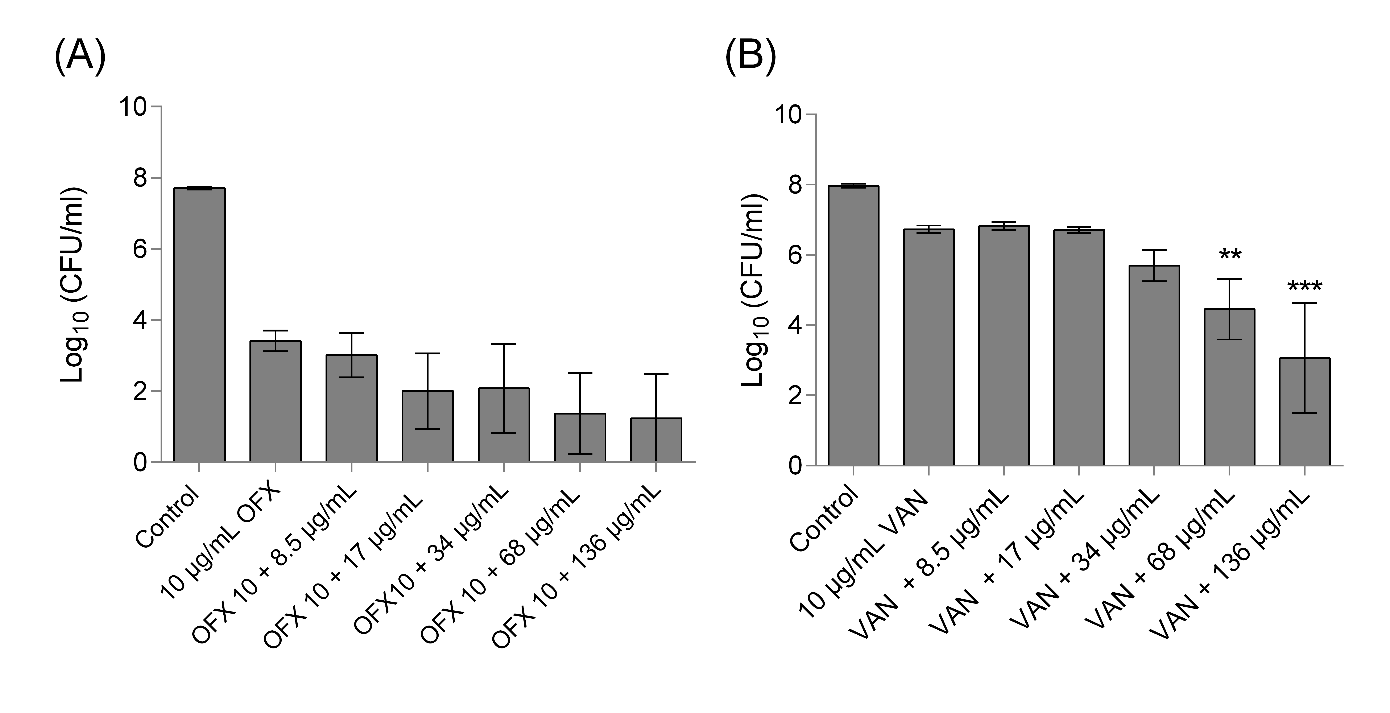


**Figure S2: Effect of conventional antibiotics in the treatment of bacterial biofilms.**  24 hour, mature biofilms of (A) *P. aeruginosa* and (B) *S. aureus* were treated for 5 hours with a conventional antibiotic, ofloxacin (OFX) and vancomycin (VAN) respectively, or the combination of this antibiotic with increasing concentrations of SPI009. Assessment of biofilm survival was done by CFU counting and statistical differences to the control treatment were detected by means of a one-way ANOVA (α= 0.05) with Dunnett’s correction for multiple comparisons. Data points represent the average of three independent repeats, each containing three technical repeats, ± SEM with ** P < 0.01 and *** P < 0.001. No significant decreases in biofilm survival were observed for the combination therapy, as compared to treatment with equal concentrations of SPI009 alone.

**Figure S3: Toxicity testing of SPI009 in *C. elegans.***  OP50 fed nematodes were monitored for 5 days in the presence of increasing concentrations of SPI009 (8.5-134 µg/mL). Controls consisted of untreated worms (negative control, black line), 2% DMSO( carrier control, red solid line) and 20% DMSO (positive control, red dotted line). Data point represent the average of at least two independent experiments, as determined by means of Kaplan-Meier survival plots. Statistical analysis was done using log-rank test which showed significant differences between the untreated control and 68 µg/mL, 136 µg/mL, 2% DMSO and 20% DMSO. No toxicity was observed for SPI009 concentrations of 8.5 µg/mL (P = 0.359), 17 µg/mL (P = 0,556) and 34 µg/mL (P = 0.094).

## Supplementary tables

**Table S1: Statistical analysis of *C. elegans* survival data.** The Kaplan-Meier method was used to analyze the obtained survival data. N represents the total number of subjects, median lifespan is considered >6 if, at the end of the experiment > 50% of the population is still alive. Kaplan-Meier curves (Figure 6) were statistically analyzed using a log-rank test to compare between treatments and control or different treatments, as indicated in the table. P_log-rank_ indicates the P-value as a result of the log-rank analysis (α = 0.05), the last column indicates significance levels after the Bonferroni correction for multiple comparisons (α = 0.01).

| **Group** | **N** | **median lifespan (days)** | **P_log-rank_** | **Bonferroni correction** |
| --- | --- | --- | --- | --- |
| un-infected | 351 | >6 |  |  |
| untreated | 165 | 4 | < 0.0001 vs un-infected | **** |
| 8.5 µg/mL SPI009 | 85 | 4 | 0.0452 vs untreated | ns |
| CIP (5 x MIC) | 132 | 6 | < 0.0001 vs untreated | **** |
| CIP + 8.5 µg/mL SPI009 | 81 | >6 | < 0.0001 vs untreated | **** |
|  |  |  | 0.0001 vs CIP | **** |
